# Evidence for Novel Pharmacological Sensitivities of Transient Receptor Potential (TRP) Channels in *Schistosoma mansoni*

**DOI:** 10.1371/journal.pntd.0004295

**Published:** 2015-12-11

**Authors:** Swarna Bais, Matthew A. Churgin, Christopher Fang-Yen, Robert M. Greenberg

**Affiliations:** 1 Department of Pathobiology, School of Veterinary Medicine, University of Pennsylvania, Philadelphia, Pennsylvania, United States of America; 2 Department of Bioengineering, School of Engineering and Applied Sciences, University of Pennsylvania, Philadelphia, Pennsylvania, United States of America; Swiss Tropical and Public Health Institute, SWITZERLAND

## Abstract

Schistosomiasis, caused by parasitic flatworms of the genus *Schistosoma*, is a neglected tropical disease affecting hundreds of millions globally. Praziquantel (PZQ), the only drug currently available for treatment and control, is largely ineffective against juvenile worms, and reports of PZQ resistance lend added urgency to the need for development of new therapeutics. Ion channels, which underlie electrical excitability in cells, are validated targets for many current anthelmintics. Transient receptor potential (TRP) channels are a large family of non-selective cation channels. TRP channels play key roles in sensory transduction and other critical functions, yet the properties of these channels have remained essentially unexplored in parasitic helminths. TRP channels fall into several (7–8) subfamilies, including TRPA and TRPV. Though schistosomes contain genes predicted to encode representatives of most of the TRP channel subfamilies, they do not appear to have genes for any TRPV channels. Nonetheless, we find that the TRPV1-selective activators capsaicin and resiniferatoxin (RTX) induce dramatic hyperactivity in adult worms; capsaicin also increases motility in schistosomula. SB 366719, a highly-selective TRPV1 antagonist, blocks the capsaicin-induced hyperactivity in adults. Mammalian TRPA1 is not activated by capsaicin, yet knockdown of the single predicted TRPA1-like gene (SmTRPA) in *S*. *mansoni* effectively abolishes capsaicin-induced responses in adult worms, suggesting that SmTRPA is required for capsaicin sensitivity in these parasites. Based on these results, we hypothesize that some schistosome TRP channels have novel pharmacological sensitivities that can be targeted to disrupt normal parasite neuromuscular function. These results also have implications for understanding the phylogeny of metazoan TRP channels and may help identify novel targets for new or repurposed therapeutics.

## Author Summary

Schistosomes, infect hundreds of millions of people worldwide, causing schistosomiasis, a disease with devastating effects on human health and economic development. Despite the prevalence of this disease, there is only a single drug, praziquantel, available for treatment and control. Praziquantel is effective, but has limitations, and reports of drug resistance lend increased urgency to development of new treatments. Ion channels are critical components of animal neuromuscular systems, and have proven to be excellent targets for drugs against parasitic worm infections. TRP ion channels, which play important roles in sensory functions, have received little attention in parasitic helminths. One class of TRP channels, TRPV, is activated by capsaicin. However, schistosomes do not appear to contain any TRPV channels. Nonetheless, we find that they are highly sensitive to capsaicin and similar compounds, responding by dramatically increasing their motor activity. Unexpectedly, suppressing expression of a different schistosome TRP channel, TRPA1, which in mammals is not sensitive to capsaicin, almost completely eliminates this response. Thus, it appears that the pharmacology of schistosome TRP channels differs from that of host mammalian channels, a characteristic that might be exploitable for development of new antischistosomal drugs.

## Introduction

Trematode flatworms of the genus *Schistosoma* cause schistosomiasis, a tropical parasitic disease that affects hundreds of millions globally [1,2], causing severe morbidity, compromised childhood development, and an estimated 280,000 deaths annually [3–5]. There is no vaccine, and treatment and control depend almost entirely on a single drug, praziquantel (PZQ) [6–8], which, though indispensable, has significant limitations, including reduced effectiveness against immature worms [9,10]. Field isolates exhibiting reduced PZQ susceptibility have been reported, and PZQ resistance can be experimentally induced [10–12], suggesting that reliance on a single treatment for a disease of this scope may be particularly dangerous.

Ion channels are membrane proteins that form a gated pore, allowing ions to flow by diffusion down the electrochemical gradient established across cell membranes. They are vital for normal functioning of the neuromusculature, as well as for other cell types, and are validated and outstanding therapeutic targets [13,14]. Indeed, the majority of current anthelmintic drugs target ion channels of the parasite's neuromuscular system [15–17], and there is increasing evidence that PZQ itself acts on voltage-gated Ca^2+^ channels [18,19]. To date, however, only a small subset of parasite ion channels has been investigated for potential targeting by new anthelmintics. One almost entirely unexplored group of schistosome (and other parasite) ion channels is the transient receptor potential (TRP) channel family [20].

TRP channels are non-selective cation channels that display an extraordinary diversity of functions and activation mechanisms [21,22]. Indeed, a single TRP channel can be activated through different, seemingly unrelated, mechanisms. TRP channels were initially discovered and characterized in *Drosophila*, with later identification of ~30 vertebrate isoforms. Though the full array of physiological functions of these channels is only gradually becoming clear, one unifying theme appears to be their key role in responding to all major classes of external stimuli (eg, light, sound, chemicals, temperature, and touch). The ability of TRP channels to transduce these signals depends largely on their role in modulating intracellular Ca^2+^ concentrations [23]. The huge potential of TRP channels as therapeutic targets has recently been extensively reviewed [24].

In addition to diverse activation mechanisms, TRP channels also show differences in ion selectivity, structure, and tissue distribution [25]. Based on sequence similarity, however, TRP channels fall into several subfamilies [21]. Mammalian types include TRPC, TRPM, TRPA, TRPV, TRPML, and TRPP. These classes, as well as others (TRPN, TRPVL), are found throughout the animal kingdom. A small subset of TRP channels are also expressed in protists, including protozoan parasites [26,27]. Representatives of five metazoan subfamilies, including TRPV, appear in the choanoflagellates [27], the unicellular common ancestors to metazoans, indicating that most classes of TRP channels emerged prior to the appearance of multicellular animals [28,29]. Schistosomes contain a wide diversity of TRP channels, but were reported to lack any predicted TRPV homologs [20,26]. Searches of current schistosome genome databases [30–32] confirm this finding.

As noted, TRP channels are often polymodal, responding to multiple stimuli. For example, TRPA1 and TRPV channels (as well as others) can be thermosensitive [33], but are also activated by chemical and mechanical stimuli. Thus, TRPV1, the mammalian vanilloid receptor, is sensitive to heat (>43°C), pH, and inflammatory factors; it is also activated with high potency by capsaicin [34–36], an active ingredient in chili peppers. Capsaicin and related compounds are selective for TRPV1; other members of the TRPV channel subfamily do not appear to respond to capsaicin [37,38], and differences in capsaicin sensitivity between different vertebrate species have been localized to a few amino acid residues in the S3 and S4 domains of the TRPV1 sequence [39–43].

Many invertebrates have genes for single or multiple TRPV-like channels, although the mammalian TRPV subtypes, including the capsaicin-sensitive TRPV1, are found only in the vertebrates [21]. Nonetheless, a few invertebrates have been reported to exhibit some sensitivity to capsaicin. Lophotrochozoans such as molluscs [44–46] and leeches [47] show cellular activation and avoidance behaviors in response to relatively high concentrations (>100 μM) of capsaicin, and capsaicin-like compounds inhibit zebra mussel (*Dreissena polymorpha*) macrofouling at micromolar concentrations [48]. In contrast, ecdysozoans such as *Drosophila* [49] and *Caenorhabditis* [50] do not exhibit acute capsaicin avoidance behaviors, though capsaicin appears to enhance thermal avoidance behavior in *C*. *elegans* [51], and *Drosophila* has been reported to exhibit a preference for the compound [49]. Interestingly, the planarian *Dugesia dorotocephala*, which, like *S*. *mansoni*, is also a platyhelminth, shows no detectable response to 10 μM capsaicin, though it does respond with increased locomotor activity to the TRPM8 agonist icilin [52]. On the other hand, oil extracts of the leaves and fruit of a Brazilian species of pepper (*Capsicum annuum*), which likely contain capsaicin, appear to have potent effects against *S*. *mansoni* cercariae, killing 90–96% within 15 min [53]. However, since the *S*. *mansoni* genome predicts no representatives of the TRPV channel subfamily [20,26] it would be reasonable to expect that schistosomes would be unresponsive to capsaicin and other TRPV1 channel modulators.

TRPA is another TRP channel subfamily, with one member, TRPA1, in mammals. TRPA1 channels are non-selective cation channels characterized by a large group of ankyrin repeats in the N- terminal domain. TRPA channels, like TRPV channels, transduce noxious thermal, mechanical, and nociceptive signals, and also mediate chronic itch [reviewed in 54]. Members of the TRPA1 clade of TRPA channels act as chemosensors for a wide variety of pungent irritants, most notably electrophilic compounds such as allyl isothiocyanate (AITC), found in mustard oil [54,55]; neither human [56] nor mouse [57] TRPA1 is activated by capsaicin. TRPA1 channels are found in a variety of organisms, and the structure of the channel has recently been reported [58]. The sub-family is represented by a single gene in *S*. *mansoni* [20,26], which we have named SmTRPA, coding for a protein with eight predicted N-terminal ankyrin repeats.

Here, we show that, though they lack a TRPV homolog, *S*. *mansoni* are sensitive to capsaicin, which elicits dramatic hyperactivity at 10^−5^ M concentrations. A TRPV1 competitive antagonist blocks these effects. Surprisingly, essentially the entire response to capsaicin is eliminated by using RNA interference to suppress SmTRPA expression in adult worms. The effect of SmTRPA knockdown on capsaicin sensitivity appears to be specific; knockdown of SmTRPA has no impact on 5-hydroxytryptamine (5-HT; serotonin)-elicited hyperactivity. Our results suggest that in schistosomes and perhaps other platyhelminths, TRPA1 channels may exhibit novel pharmacological sensitivities, opening the possibility for selective targeting and perhaps providing clues to the phylogenetic relationship of these TRP channel sub-families.

## Methods

### Ethics statement

This study was carried out in strict accordance with the recommendations in the Guide for the Care and Use of Laboratory Animals of the U.S. National Institutes of Health. Animal handling and experimental procedures were undertaken in compliance with the University of Pennsylvania's Institutional Animal Care and Use Committee (IACUC) guidelines (Animal Welfare Assurance Number: A3079-01). The IACUC approved these studies under protocol number 804217.

### Reagents

Capsaicin and SB 366791 were from Cayman Chemical (Ann Arbor, MI), resiniferatoxin (RTX) was from LC Laboratories (Woburn, MA), and allyl isothiocyanate (AITC) and 5-hydroxytryptamine (serotonin) were from Sigma-Aldrich (St. Louis, MO). Reagents were dissolved in dimethyl sulfoxide (DMSO) for stock solutions and then diluted to an appropriate concentration in culture media. All oligonucleotides and siRNAs were from Integrated DNA Technologies (IDT, Coralville, IA).

### Isolation of schistosomes

Female Swiss Webster mice infected with *S*. *mansoni* (NMRI strain) were provided by the Schistosomiasis Resource Center for distribution by BEI Resources, NIAID, NIH (*S*. *mansoni*, Strain NMRI—exposed Swiss Webster mice, NR-21963). Adult worms were perfused at 6–7 weeks post infection as described [59]. Perfused worms were maintained in RPMI (Life Technologies, Inc., Grand Island, NY) plus 10% FBS (Sigma-Aldrich) and 1% penicillin/streptomycin (Sigma-Aldrich) at 37°C and 5% CO_2_. Schistosomula were obtained by *in vitro* transformation of cercariae [59] and maintained in the same culture conditions as adults.

### Cloning of SmTRPA

There is a single gene in the *S*. *mansoni* genome (Smp_125690) predicted to code for a TRPA channel, which we have dubbed SmTRPA. However, the coding region found in the database for SmTRPA appears to be incomplete. Though the predicted SmTRPA protein contains the series of ankyrin repeats that define TRPA channels, a large portion of the channel domain itself is missing. We used 5' and 3' RACE on *S*. *mansoni* RNA to obtain a complete coding region. The total RNA we used as template was from adult male worms, and was provided by the Biomedical Research Institute (distributed by BEI Resources, Manassas, VA). 500 ng of this RNA was used in the SMARTer RACE 5'/3' Kit (Clontech, Mountain View, CA) according to the manufacturer's instructions, with the following gene-specific primers: for 3' RACE, 5' TCAAGGTCCAGGAATCAGAACAGTCCTA 3' and the nested primer 5' CGTGGGGCTTCTGCATTAGAACGTGAT 3'; for 5' RACE, 5' CGTTCTAATGCAGAAGCCCCACGTA 3' and the nested 5' TAGGACTGTTCTGATTCCTGGACCTTGA 3' (both with the sequence 5' GATTACGCCCAAGCTT 3' at their 5' ends to facilitate In-Fusion cloning to the Clontech pRACE vector, as per the manufacturer's instructions), to obtain the full-length coding sequence that now contains the full channel domain. We also used spliced leader primers [60] to verify the 5' end. The 3' end of the cDNA coding region is extended 993 bp beyond base 1936 of the predicted 3' end of Smp_125690. This additional sequence includes the remaining transmembrane regions of the channel domain. In contrast, the 5' start of the coding region that was predicted in the genome database is the same as that found by us in the cDNA following RACE. However, in addition to the 3' extension, the cDNA also contains three insertions not found in the predicted amino acid sequence in the genome database. These include a ~250 bp (82 aa) insertion following base 319 (of the genomic predicted sequence), a ~200 bp (67 aa) insertion following base 712, and a 108 bp (36 aa) insertion following base 1764. The full-length coding sequence was amplified by PCR using high-fidelity Q5 DNA polymerase (NEB), using terminal primers with 5' overlapping vector sequence, cloned into the EcoRV site of the pcDNA3.1-zeo vector (Life Technologies, Inc.) by In-Fusion cloning (Clontech), and re-sequenced.

### Exposure of schistosomes to pharmacological compounds

Adult worms were first placed individually in standard schistosome medium in single wells of a 24-well plate for 15 min. During this period, control motility readings were taken (see below). TRP channel modulators (or DMSO carrier) were then added to the medium to appropriate final concentrations, and motility measured again over the course of another 15 min. Each worm thus served as its own control. Serotonin (5-HT) at 40 μM was used as a control for increased motility and 500 nM PZQ served as a control for reduced motility (i.e., paralysis). Vehicle controls included 0.1% DMSO. *In vitro*-transformed schistosomula were exposed in a similar manner, but contained several worms per well. For inhibition studies with SB 366791, adult males or female worms were first imaged for their control motility, then pre-incubated for 30 min in antagonist (with a 15-min measurement of motility), followed by addition of capsaicin and imaging for measurement of motility again, as described above.

### Measurement of schistosome motility

For measurement of adult worm motility, we adapted an imaging system and software used for monitoring of *C*. *elegans* locomotor activity [61]. The imaging system consists of a USB monochrome camera (2592 x 1944 pixel resolution, DMK 72BUC02, The Imaging Source, Charlotte, NC), a 12.5 mm, f/1.4 fixed-focus lens (HF12.5SA-1, Fujinon, Fujifilm, Valhalla, NY), a red flexible LED strip for illumination, and other mechanical components, as described [61]. Images were acquired for the entire 24-well plate, each well of which contained a single worm, over the course of 15 min at 15 frames per second, and saved to the hard drive in BMP format using Image Capture software. After completion of image acquisition we used custom MATLAB software to calculate motility for each worm by measuring absolute differences in gray scale values between consecutive images within each region of interest (eg, each well), a metric that is sensitive to any type of movement [61]. For each pair of successive frames, we summed all pixels in which a change in intensity greater than a threshold occurred, yielding a measurement of the amount of motion within the region of interest between the two frames. Using these values, we calculated an average change in pixel values per frame across the 15-minute window for each worm, and normalized that value to 100 for the control worms.

For schistosomula, we used a Leica stereomicroscope with Qicam Fast 1394 camera (Qimaging) and Q-Image capture software to create videos at 2 frames per second over a 2.5-minute time span (300 frames). These video recordings were then used to analyze motility of individual worms using MaxTraq-Lite+ motion analysis software (Innovision Systems, Columbiaville, MO), as described [62]. We measured the distance between the ends of individual worms as an estimate of body length every 3 frames, and then calculated the change in distance between each measurement (as the worm moves more rapidly, body length will change with increased frequency).

### RNA interference

Knockdown of RNAs encoding the single *S*. *mansoni* homologs of TRPA1 (SmTRPA1, Smp_125690) and TRPM7 (SmTRPM7, Smp_035140), and one of the three *S*. *mansoni* TRPM3-like sequences (SmTRPM3a, Smp_165170) in adult worms was as described [63,64]. The luciferase siRNA used as a control (Silencer Firefly Luciferase, GL2+GL3, Life Technologies, Inc.) shows no significant similarity to any sequences from the *S*. *mansoni* gene database. siRNAs against the *S*. *mansoni* TRP channel were designed using the SciTools software suite from IDT. The target sequences were: SmTRPA siRNA, 5'-GAGTTGAAACGTGAAGAGTTATTAATT-3', which maps to bp 1561–1587 of the predicted coding region (in the *S*. *mansoni* database) of SmTRPA (Smp_125690); SmTRPM7 siRNA, 5'-ACCTGATGAAGAGAATAGTAATTTGAA-3', corresponding to bp 2792–2818 of SmTRPM7 (Smp_035140); and 5'-GGAGTGCATACCAATGCATTTGT-3', corresponding to bp 2128–2152 of SmTRPM3a (Smp_165170).

Following overnight incubation in schistosome medium, adult worms (5 males and 5 females) were placed in a 0.4 cm electroporation cuvette (USA Scientific, Ocala, FL) containing 50 μl media plus 5 μg of SmTRP or control luciferase siRNAs (IDT). Worms were electroporated in this solution with a 20 ms square-wave pulse of 125 volts (Pulser XCell, BioRad, Hercules, CA). Following electroporation, worms were incubated in schistosome medium for 2 days, then sorted into a single male or female per well of a 24-well plate These worms were tested for sensitivity to capsaicin and other compounds as described above.

### Real-time RT-PCR

Real-time RT-PCR was used to measure levels of knockdown by RNAi. Total *S*. *mansoni* RNA was extracted from adult worms using Direct-Zol RNA Mini Prep (ZYMO Research, Irvine, CA) according to the manufacturer's instructions. qRT-PCR was performed using the Brilliant II SYBR green qRT-PCR Master kit (Agilent Technologies, Santa Clara, CA) on an Applied Biosystems 7500 according to the manufacturer's recommendations and as described [62]. Primers used for the amplification of 18S ribosomal RNA have been described [62]. Primers used for amplification of SmTRPA1 were: TRPA FWDSET1 (5′-TCGTCTTGGAGCAAATCCTAAT-3′) and TRPA REVSET1 (5′-CTGGTAGGACTGTTCTGATTCC-3′). Primers used for amplification of SmTRPM7 were: TRPM7 FWDSET1 (5′-GAGAACCCAGTCCAGGTTTAAG-3′) and TRPM7 REVSET1 (5′-GCTAACATCGGTCGTATCCATT-3′). Data were analyzed using the 2^−ΔΔ^C_t_ method [65] to determine the relative expression ratio between targets (TRP channels) and reference genes (18S RNA).

### Statistics

**Fig 1:**
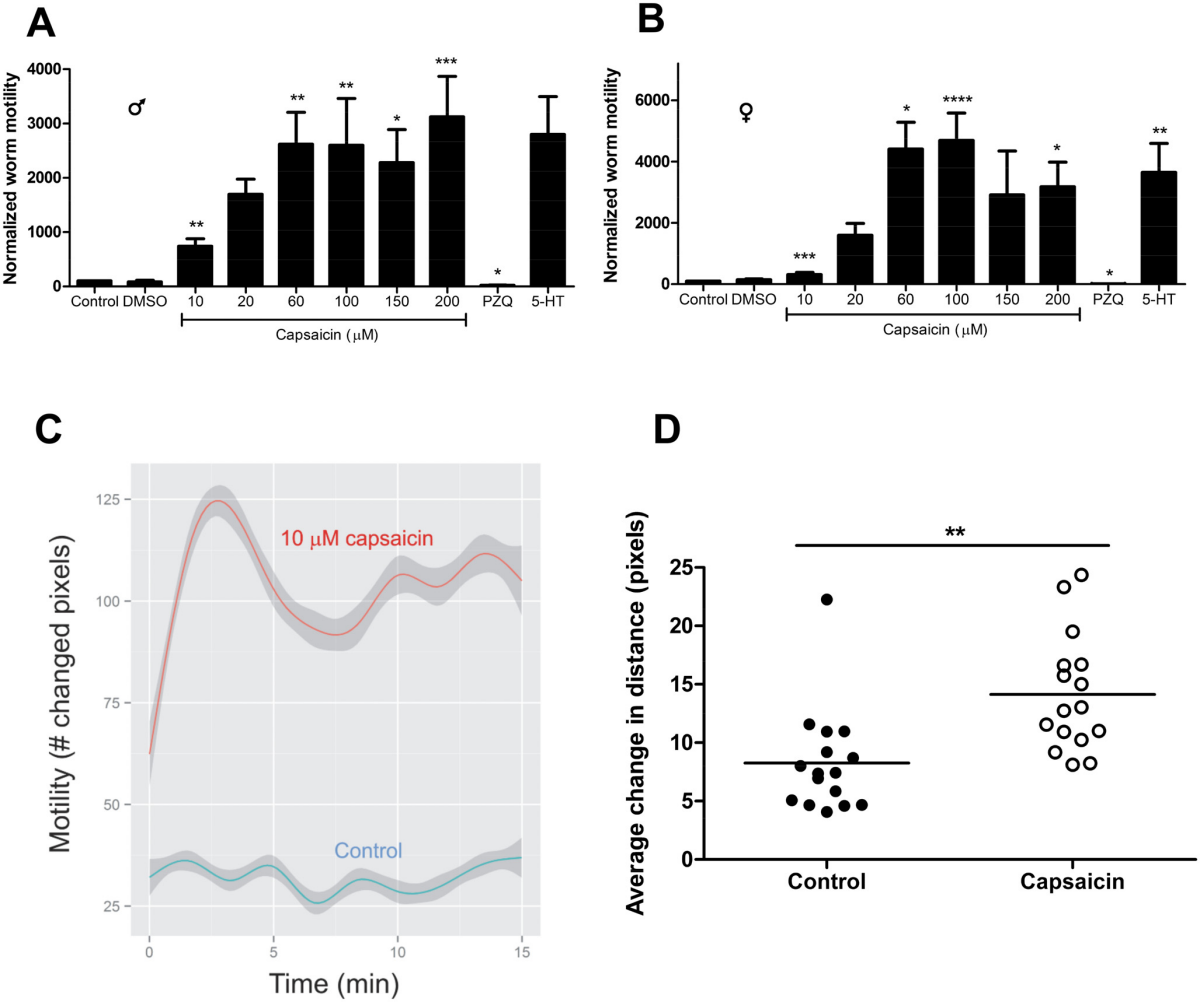
Capsaicin induces hyperactivity in *S*. *mansoni* adults and schistosomula. Worm motility was measured initially in standard schistosome media over the course of 15 min to set a baseline for individual worms. Worms were then incubated in media containing capsaicin (or other compounds) at the concentrations shown and worm motility measured for another 15 min and normalized to a value of 100 for the "no-drug" control period for each worm. Thus, each worm served as its own control. Assessments of motility were made by measuring absolute differences in gray scale values between consecutive images, as described in Materials and Methods. Controls for both hyperactivity (40 μM 5-HT) and paralysis (500 nM PZQ) were included to test whether our system for measuring activity is robust. **A.** Adult male responses to capsaicin. Histogram of normalized motility vs. capsaicin concentration, along with controls. n = 5–10. **B.** Adult female responses to capsaicin, as in A. n = 5–7. **C.** Capsaicin-induced worm hyperactivity is sustained for at least 15 min. Shown are data for male worms in 10 μM capsaicin vs. control, n = 6. **D.** Capsaicin (20 μM) increases motility in schistosomula. Activity of *in vitro*-transformed schistosomula was measured initially in standard schistosome media and then following addition of 20 μM capsaicin. Activity was measured using the distance algorithm of MaxTraq-Lite+, as described [62]. n = 16, across 4 independent experiments. *, **, ***, ****, P<0.05, P<0.01, P<0.001, P<0.0001 respectively, paired t-test vs. control conditions (prior to normalization). All data are presented as means ± SEM.

Data were analyzed with GraphPad Prism or Microsoft Excel, expressed as arithmetic means ± SEM, and tested for statistical significance using statistical tests noted in the figure legends. In the drug response studies, each worm served as its own control, and we therefore compared means using paired t-tests (on the raw data, prior to normalization). The time course of motility (Fig 1C) was analyzed and plotted using R v. 3.13, ggplot2 package. For the knockdown experiments, we compared motility between knockdown and control worms in each of the drug concentrations; in this case, differences between means were therefore tested using unpaired t-tests.

## Results

### The TRPV1 activators capsaicin and RTX induce hyperactivity in schistosomes

Capsaicin, a vanilloid compound that is an active ingredient in hot peppers, is a potent and selective activator/enhancer of TRPV1 [37]. Exposure of *S*. *mansoni* adult males or females to capsaicin produces a marked increase in worm motor activity that persists for at least 15 min. Fig 1A shows responses of male schistosomes to different concentrations of capsaicin, ranging from 10 μM to 200 μM, using an imaging system that assesses the number of pixels where differences in gray scale values occur between consecutive video frames as a measure of motility [61]. Adult males in capsaicin show a dose-dependent, saturable enhancement of motility, with a ~10-fold increase at 10 μM capsaicin and a 20-30-fold increase in concentrations of capsaicin equal to or higher than 60 μM. Controls for both hyperactivity (40 μM 5-HT) and paralysis (500 nM PZQ) indicate that this system for measuring motility is robust (Fig 1A). Adult female worms also show a dose-dependent enhancement of motility in 10–200 μM capsaicin (Fig 1B). As with males, the response appears to saturate at capsaicin concentrations of 60 μM and higher. Attempts to measure effects of capsaicin on paired male and female worms (the more biologically relevant condition in which schistosomes reside within the host) were not possible, as worm pairs rapidly and invariably separate when exposed to capsaicin (S1 Video), perhaps indicative of a nociceptive response. The effects of 10 μM capsaicin on worm motility are sustained through a 15-minute time period, as shown in Fig 1C.

Capsaicin (20 μM) also elicits increased activity in *in vitro*-transformed schistosomula, indicating that different schistosome stages are susceptible to vanilloid compounds that target the TRPV1 channel in mammals. (Fig 1D). Although the change in activity in schistosomula does not appear to be as striking as in adult worms, the two sets of data were obtained using different assays of motility, and direct comparisons are not applicable. Interestingly, consistent with previous findings showing cercaricidal effects of an oil extract from the pepper plant *C*. *annuum* [53], we find that either 60 μM or 100 μM capsaicin effectively abolishes cercarial swimming. The cercariae retain their tails, but instead of swimming, remain largely in one place, moving in a fashion reminiscent of schistosomula (see S2 Video and S3 Video).

**Fig 2:**
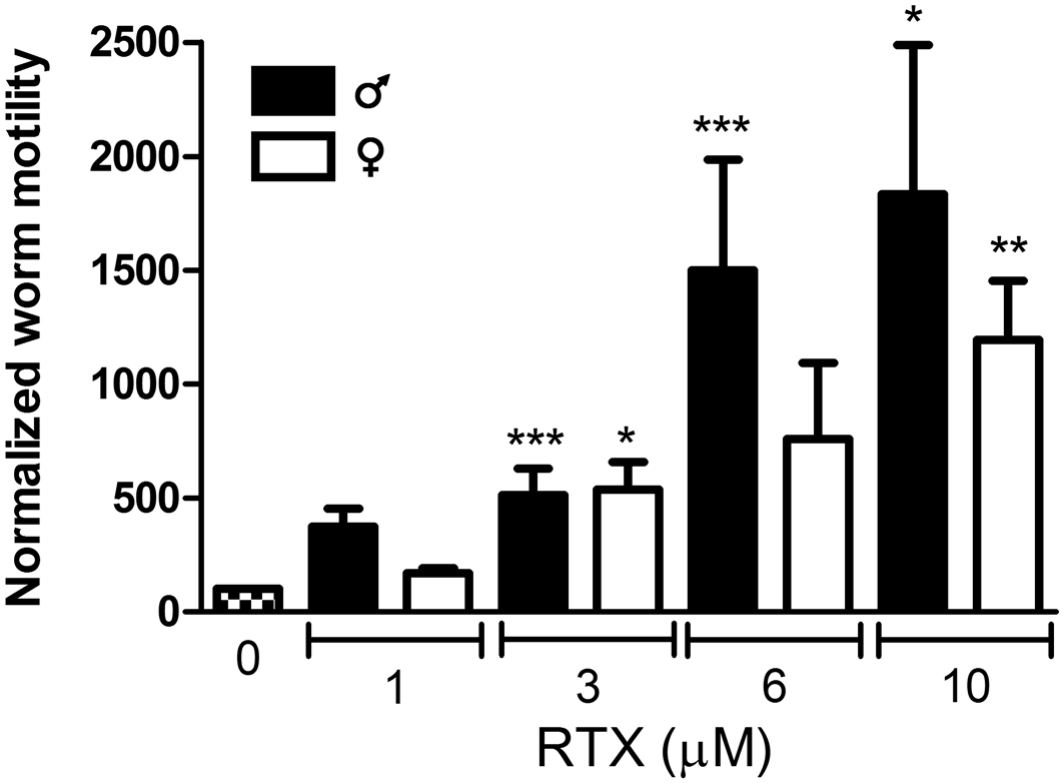
RTX, a highly specific and potent TRPV1 activator, increases motor activity in adult schistosomes. Normalized motility of adult *S*. *mansoni* males (black bars) and females (white bars) exposed to 1–10 μM RTX was measured as described in Fig 1. *, **, ***, P<0.05, P<0.01, P<0.001 respectively, paired t-test vs. Controls (prior to normalization). All data are presented as means ± SEM, n = 5–14 for males, 4–6 for females.

Resiniferatoxin (RTX), a toxin found in the dried latex of *Euphorbia resinifera*, acts as an analog of capsaicin, potently activating the TRPV1 channel [37] and binding to the TRPV1 vanilloid binding site [41]. Like capsaicin, RTX (1–10 μM) increases motility in both male and female adult schistosomes, with significant effects seen at concentrations as low as 3 μM (Fig 2).

### The competitive TRPV1 antagonist SB 366791 blocks responses of *S*. *mansoni* adults to capsaicin

**Fig 3:**
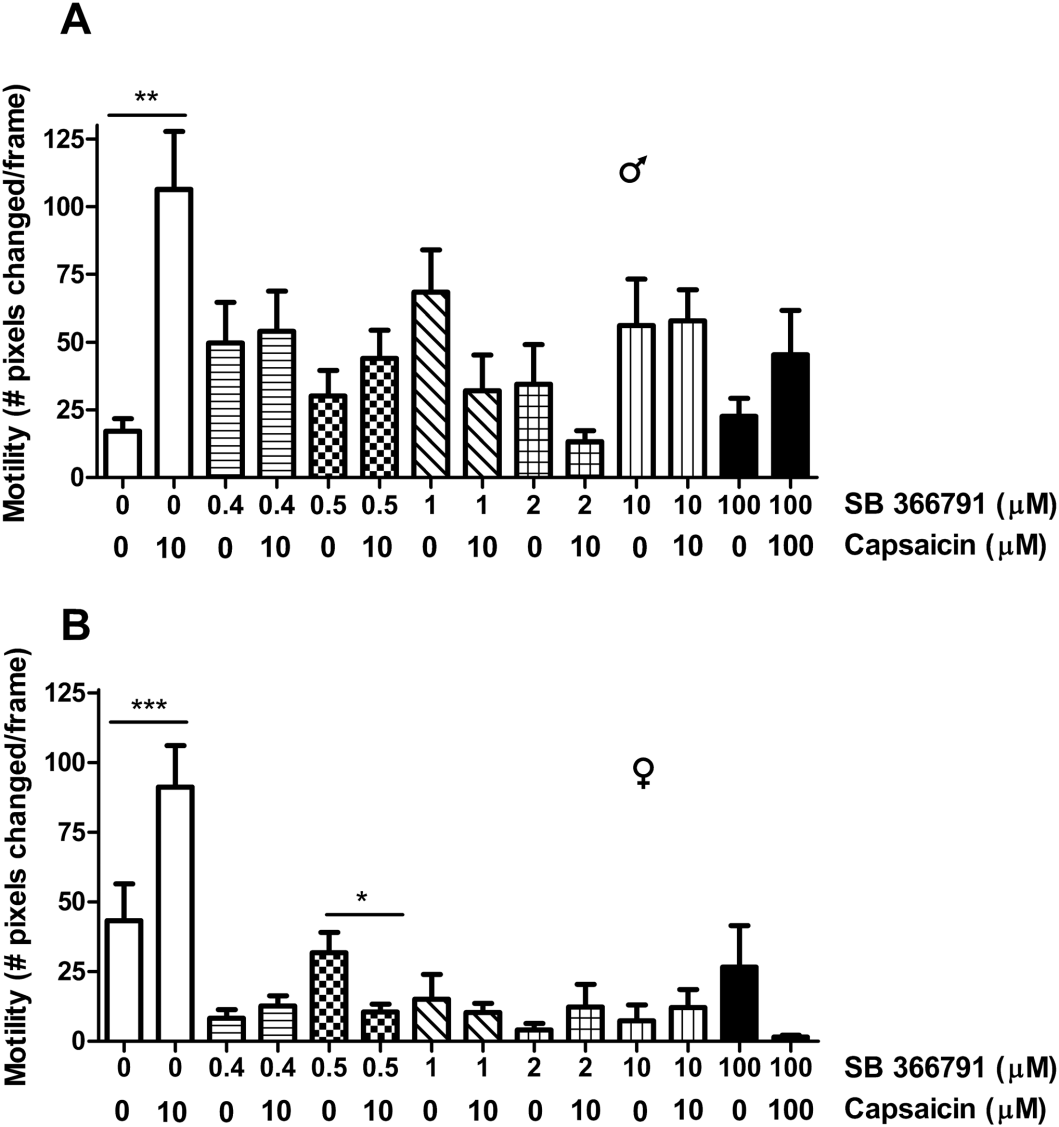
SB 366791, a potent, highly-selective TRPV1 antagonist, inhibits capsaicin-induced increased motility in *S*. *mansoni* adults. Motility was measured as in Fig 1 in individual males (**A**) and females (**B**). White bars show the significant increase in worm motility in response to 10 μM capsaicin in the absence of SB 366791. Differently patterned bars designate worm motility in 0 vs. 10 μM capsaicin following pre-incubation in (and co-exposure to) SB 366791 at concentrations of 0.4 μM (horizontal lines), 0.5 μM (checkerboard), 1 μM (diagonal lines), 2 μM (squares) or 10 μM (vertical lines). The pair of black bars shows responses of worms to 100 μM capsaicin in the presence of 100 μM SB 366791 (see Methods for details). There is a significant increase in worm motility in response to capsaicin only in the absence of SB 366791. *, **, ***, P<0.05, P<0.01, P<0.001 respectively, paired t-tests vs. no-capsaicin controls. n = 4–8.

We tested the potent, highly-selective [66] competitive TRPV1 antagonist SB 366791 to determine whether it blocks worm responses to capsaicin. SB 366791 has been shown to have no effect on AITC-evoked (ie, TRPA1-mediated) responses in mice [67], although a recent report [68] indicates that 10 μM SB 366791 can antagonize responses to AITC in particular leech neurons. As shown in Fig 3, the significant increases in motility induced by capsaicin do not occur when worms are pre-incubated in concentrations of SB 366791 ranging from 400 nM to 100 μM. Despite indications in Fig 3 that SB 366791 may have motility effects on its own, particularly in females, direct tests (S1 Fig) show that the compound exhibits no significant effect on worm motility. These results indicate that in schistosomes, which appear to lack TRPV-like channels, the capsaicin receptor has pharmacological sensitivities that are similar to those of mammalian TRPV1 channels.

### Knockdown of SmTRPA attenuates *S*. *mansoni* response to capsaicin

The *S*. *mansoni* genome predicts no TRPV homologs. We therefore initiated experiments to determine if other *S*. *mansoni* TRP channels might be mediating the parasite's response to capsaicin. We first examined SmTRPA, the only TRPA1-like gene predicted in the *S*. *mansoni* genome. TRPA channels appear to be most closely related phylogenetically to TRPV channels, are frequently co-expressed with TRPV channels, and often serve analogous functions and transduce signals (nociceptive, thermal, inflammatory, etc.) similar to those transduced by TRPV channels [28,69–71]. Mammalian TRPA1 does not respond to concentrations of capsaicin that activate TRPV1 channels [56,57].

**Fig 4:**
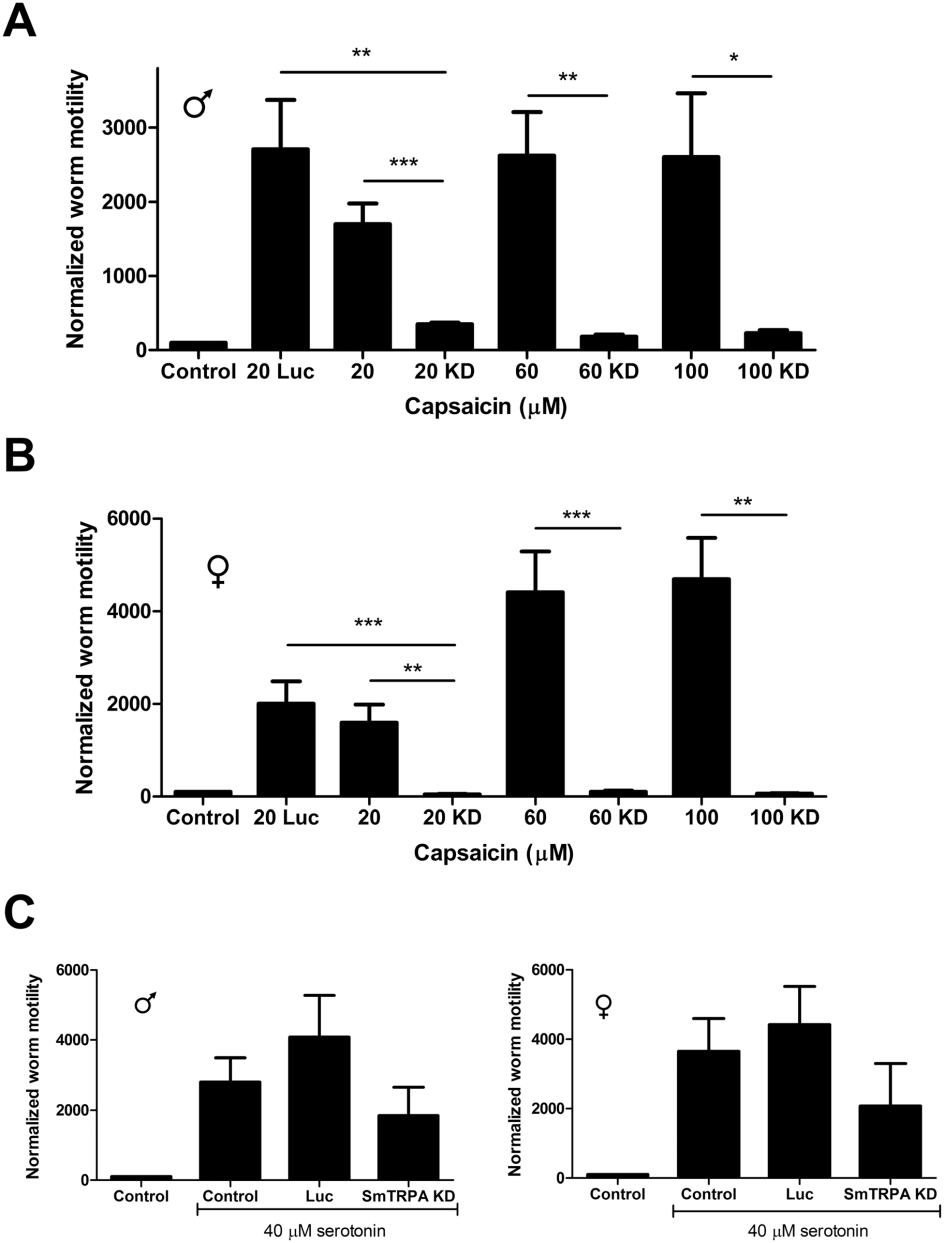
Knockdown of SmTRPA eliminates schistosome response to capsaicin. Comparison of capsaicin-dependent worm motility between normal adult worms (eg, no electroporation or knockdown) and those electroporated with 5 μg SmTRPA siRNA (KD). (**A**) Male worms. (**B**) Female worms. Capsaicin concentrations are shown. Those bars labeled with only the capsaicin concentration (20, 60, 100) on the X axis designate normalized motility following exposure to that concentration of capsaicin in worms that have not been subjected to knockdown. Those showing the capsaicin concentration plus "KD" (20 KD, 60 KD, 100 KD) designate capsaicin-induced normalized motility in worms subjected to knockdown of SmTRPA. Also shown are "Control" worms (no knockdown, no capsaicin) and worms electroporated with 5 μg luciferase siRNA (Luc) and exposed to 20 μM capsaicin as a control for effects of electroporation with siRNA. Worm activity was assessed as in Fig 1. *, **, ***, P<0.05, P<0.01, P<0.001 respectively, unpaired t-test vs. control worms for each capsaicin concentration. n = 5–10. **C.** Knockdown of SmTRPA (SmTRPA KD) has no significant effect on response of adult males (left) or females (right) to 40 μM serotonin. "Control", "Luc" labels are the same as in A and B. n = 4–6.

Electroporation of adult worms with SmTRPA siRNA reduced SmTRPA expression ~90% in males and ~60% in females (S2A Fig). SmTRPA knockdown significantly attenuated the response to capsaicin in both adult male and female worms. Thus, following knockdown of SmTRPA in males (Fig 4A), the response to capsaicin drops from a 14-fold increase in motility at 20 μM capsaicin to a 3.4-fold increase in worms exposed to SmTRPA siRNA. At higher capsaicin concentrations, the effects of knockdown are even more dramatic, with essentially the entire capsaicin-dependent increase in motility eliminated. In females (Fig 4B), knockdown of SmTRPA abolishes effectively the entire response to capsaicin at all concentrations tested (electroporation with a control luciferase siRNA has no significant effect on capsaicin-dependent hyperactivity in males or females). In contrast (Fig 4C), knockdown of SmTRPA expression does not affect stimulation of motility by serotonin [72], indicating that suppression of SmTRPA expression does not compromise the parasite neuromuscular system nonspecifically; these worms can still respond to agents that evoke hyperactivity through other pathways.

To further assess whether the effects of SmTRPA knockdown on capsaicin sensitivity are specific, we used RNAi to knock down -other two *S*. *mansoni* TRP channel genes: SmTRPM7 (Smp_035140), which encodes a TRPM7/M6-like protein; and one of the TRPM3-like genes (SmTRPM3a; Smp_165170) found in the *S*. *mansoni* genome. In adult male worms, knockdown of SmTRPM7 (S2B Fig) produces no significant effect on capsaicin sensitivity compared to control worms (S3 Fig). Thus, male worms with suppressed SmTRPM7 expression continue to exhibit large (>15-fold) increases in motility in response to capsaicin. On the other hand, there is some evidence for an effect of SmTRPM7 knockdown on responses of females to capsaicin, with a significant decrease from controls at 100 μM capsaicin. Nonetheless, these SmTRPM7-knockdown worms continue to exhibit a ~20-fold increase in activity when exposed to 100 μM capsaicin. A double knockdown of SmTRPA and SmTRPM7 (S2C Fig) decreases responsiveness to 20 μM capsaicin in males approximately 4-fold (P<0.001) and to 60 μM capsaicin approximately 2-fold in females (P<0.05) compared to the single SmTRPA knockdowns (S4 Fig), again indicating the possibility of some contribution of other schistosome TRP channels to capsaicin-induced hyperactivity.

Interestingly, knockdown of SmTRPM3a does produce a significant effect on capsaicin responsiveness (S5 Fig). The worms still react to the compound, but far less dramatically (~4-5-fold vs. 15-25-fold). However, in contrast to schistosomes with SmTRPA knocked down, which continue to respond to serotonin (Fig 4), worms with SmTRPM3a knocked down exhibit a significantly diminished response to serotonin (S5 Fig). These results suggest that SmTRPM3a may play a role in the ability of the schistosome neuromuscular system to respond to a variety of agents that stimulate activity. Knockdown of SmTRPM3a apparently compromises this capability, but is not specific to any particular stimulant. These worms appear to have normal basal levels of motility however.

### The TRPA1 activator AITC also induces SmTRPA-dependent hyperactivity in *S*. *mansoni* adults

**Fig 5:**
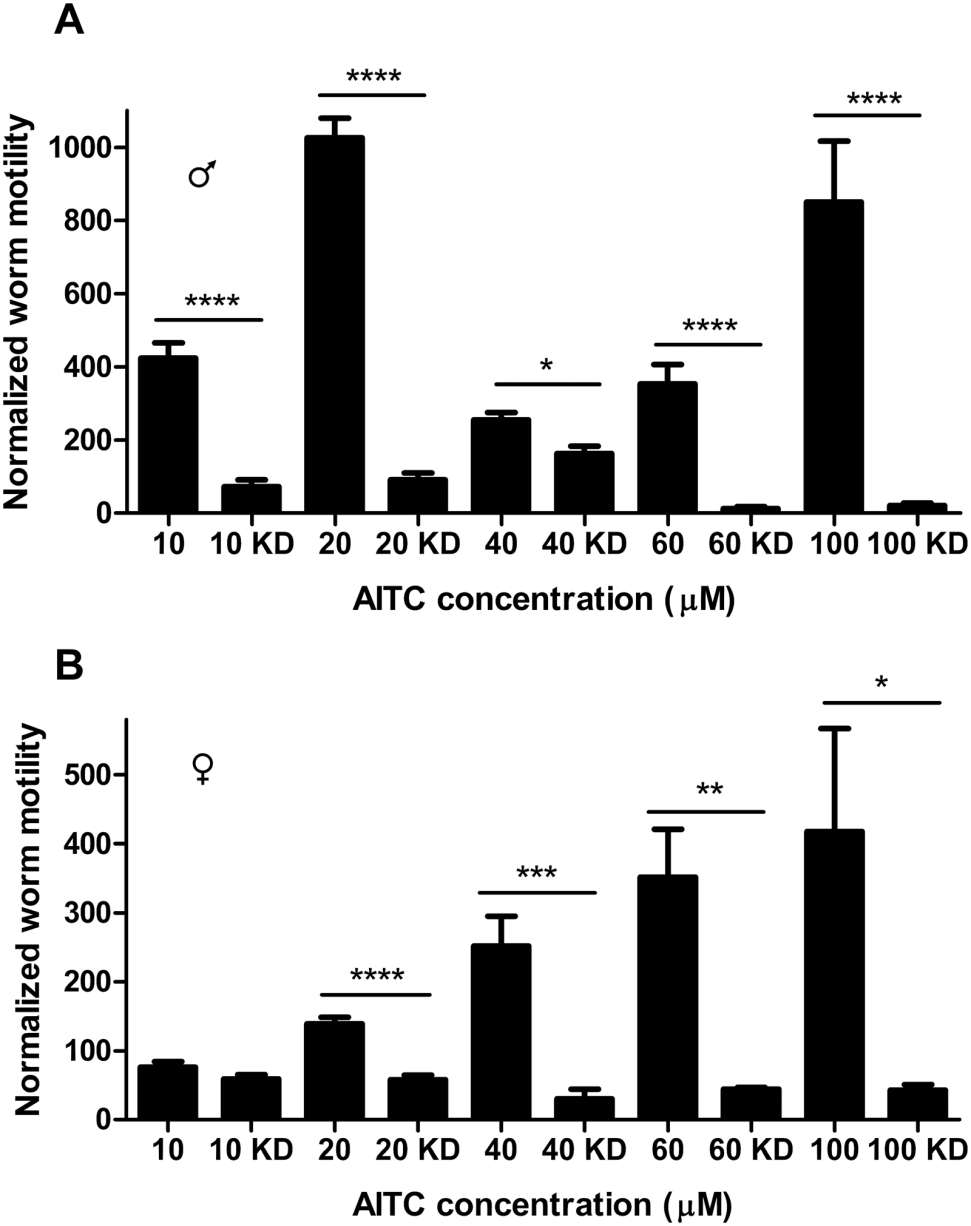
Knockdown of SmTRPA attenuates the hyperactivity elicited by the TRPA1 activator AITC. Adult males (**A**) and females (**B**) were exposed to different concentrations of AITC, and motility measured, as described in Figs 1 and 4. The AITC-dependent normalized change in motility at each AITC concentration for these control worms (not exposed to any siRNA; 10, 20, 40, 60, 100) is compared with the AITC-dependent normalized change in motility for worms with SmTRPA expression suppressed by RNAi (10 KD, 20 KD, 40 KD, 60 KD, 100 KD). *, ***, ****, P<0.05, P<0.001, P<0.0001 respectively, unpaired t-test. n = 4–8.

AITC is an organosulphur compound found in mustard and wasabi. It is one of several pungent electrophilic compounds that activate TRPA1 channels through covalent modification of cysteines [73–75]. AITC also activates/sensitizes TRPV1, though at relatively high concentrations compared with TRPA1, via a mechanism independent of cysteine modification [76–78]. Adult male and female schistosomes exposed to AITC display a significant increase in activity (Fig 5). In contrast with capsaicin, to which females exhibit more pronounced responses than males, AITC elicits substantially higher levels of hyperactivity in male worms that in females. When exposed to 10 μM AITC, males show a 4-5-fold increase in motility (Fig 5), while females show no significant change. At 20 μM AITC, motility increases approximately 10-fold in males compared to control worms, but only slightly (~1.5-fold), though significantly, in females. Interestingly, though 40 μM AITC continues to evoke increasing hyperactivity in females, producing a 2.5-fold rise, in males, 40 μM and 60 μM AITC produce a significant (P<0.0001 and P<0.001, respectively) decrease in levels of hyperactivity compared to the 20 μM concentration. Nonetheless, in males as in females, 40 μM and 60 μM AITC still elicit 2.5- and 3.5-fold increases in motility over controls, respectively. Interestingly, at 100 μM AITC, the increase in motility in males returns to the level at 20 μM AITC. It is not clear why there is a diminished response at the 40 μM and 60 μM concentrations, but it perhaps reflects a balance of increasing activation and non-selective toxicity.

We also tested the effects of suppressing SmTRPA expression on AITC sensitivity in adult worms. As with capsaicin, knockdown of SmTRPA essentially eliminates schistosome sensitivity to AITC (Fig 5), indicating that SmTRPA expression is required for parasite responsiveness to AITC.

### Identification of the open reading frame of SmTRPA

The amino acid sequence of SmTRPA (Smp_125690) predicted in the *S*. *mansoni* genome database is missing a large portion of the channel domain found in all TRP channels, and thus appears to be incomplete. We therefore cloned the full-length coding region of SmTRPA, using RACE and other protocols (see Methods), extending the predicted open reading frame 993 bp at the 3' end. The cDNA also contains inserted stretches not found in the open reading frame predicted in the genome database. This sequence has been deposited to GenBank, with accession #KT266713.

**Fig 6:**
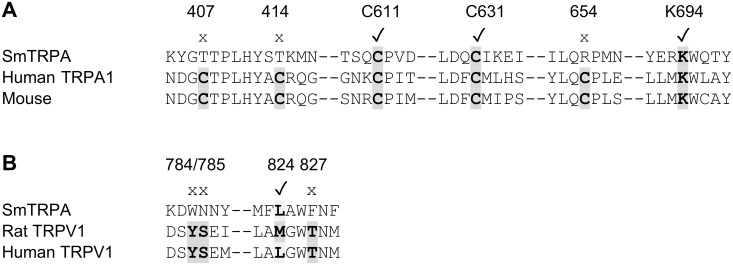
Examination of SmTRPA sequence for residues identified as important for drug activity. Alignments were made with ClustalW2 (http://www.ebi.ac.uk/Tools/msa/clustalw2/) and Swiss-Model (http://swissmodel.expasy.org/). Numbering is based on the SmTRPA amino acid sequence. SmTRPA residues that are conserved are marked with a check mark (✓) above and are in bold and shaded, and those that are not conserved are marked with an x. **A.** Cysteine residues identified in mouse (equivalent to SmTRPA 407, 414, 611) or human (611, 631, 654) as important for activity of AITC and other electrophilic compounds on TRPA1 are shown. A lysine residue (K694) appears to be critical in human TRPA1 as well, and is also shown in bold and shaded. **B.** Residues identified as important for activity of vanilloid compounds on TRPV1 are not well conserved in SmTRPA. Of 4 residues thought to be important for capsaicin and other vanilloid activity, only the methionine/leucine residue at SmTRPA residue L824 appears to be conserved.

Analysis of the SmTRPA sequence reveals that it contains many, but not all, of the residues thought to be required for AITC activity. As noted above, a wide range of electrophilic compounds such as AITC react with mammalian TRPA1 via covalent modification of cysteine residues [69,79]. The three cysteine residues in mouse TRPA1 that appear to be required for this activity are C415, C422, and C622 [75]. In human TRPA1, C619 (the equivalent of mouse C622) is also crucial, as are C639 and C663, as well as a lysine residue, K708 [74]. As shown in Fig 6A, SmTRPA contains the equivalent of the C619/622, C639, and K708 residues found in mouse and human TRPA1 proteins; C415, C422, and C663 are not conserved.

## Discussion

Ion channels are validated targets for anthelmintics, yet only a small subset of this superfamily has been characterized in parasitic helminths. Notably, TRP channels, which are critical to sensory transduction, ion homeostasis, and other cellular functions, are almost entirely unexplored in these organisms. The *S*. *mansoni* genome contains 15–20 genes predicted to code for TRP channels that represent nearly all of the major metazoan sub-families. Notably, however, there appear to be no representatives of the TRPV channel sub-family [20,26]. Indeed, we have verified the absence of TRPV-like schistosome sequences by querying schistodb.net [30], which contains current databases for the three major human schistosome species, for TRPV-like sequences, using BLAST (we have also searched genedb.org and NCBI). Consistent with previous reports [20,26], we have never found any schistosome sequences with significant similarity to a TRPV channel, other than some predicted non-TRPV proteins containing homologous ankyrin domains. Though a lack of TRPV channels is unusual, it does appear to occur in some other metazoans as well [28]. In this communication, we show that the *S*. *mansoni* TRPA1 channel has seemingly taken on at least some of the pharmacological characteristics of the mammalian TRPV1 channel. Schistosomes show sensitivity to capsaicin and RTX, selective activators of TRPV1 in mammals [37,38,56,57], as well as the highly-selective TRPV1 antagonist SB366791, but lose capsaicin sensitivity when SmTRPA expression is suppressed. Functional characterization of SmTRPA, either in a heterologous system, or in dissociated schistosome tissues [80] could confirm this novel pharmacology as well as provide opportunities to define other pharmacological and biophysical properties of this channel. It would also be interesting to determine if TRPA1 channels in other metazoans that lack TRPV channels show TRPA-dependent sensitivity to TRPV1 ligands as well.

The question of which structural features of SmTRPA might be responsible for capsaicin sensitivity is intriguing. Particular amino acid residues appear to be required for binding of vanilloid ligands to TRPV1 channels. These include Y511 and S512 in transmembrane segment 3, and M/L547 and T550 in transmembrane segment 4 [39–41]. Examination of SmTRPA aligned with rat and human TRPV1 and other TRPA1 sequences (Fig 6B) reveals that, with one exception, it does not appear to share those residues. The exception is M/L547, for which the equivalent residue in SmTRPA (and *Drosophila* TRPA1) is a leucine (L824), as it is in human (and rabbit) TRPV1. A switch from leucine to methionine at this position in human TRPV1 is associated with a 20-30-fold increase in affinity for RTX [39–43].

Our results raise interesting questions regarding the relationships between different TRP channels, including how these channels have evolved and the level of fluidity between the different TRP channel types. They also could have important implications for development of antischistosomal therapeutics. The parasite neuromuscular system is the site of action for many anthelmintics. More specifically, ion channels, required for normal neuromuscular function, have proven to be outstanding anthelmintic targets. Nonetheless, only a few ion channel families have been investigated in detail in parasitic helminths; TRP channels notably represent one of these ion channel families that has remained largely unexplored. Our results show that expression of schistosome SmTRPA is required for vanilloid-dependent (and AITC-dependent) dysfunction (hyperactivation) of the parasite neuromuscular system (though not serotonin-dependent activation), perhaps providing an entrée for a novel set of targets for new or repurposed antischistosomals. SmTRPM3a, which appears to play a broader role in stimulation of schistosome motility, may represent another, more general point of attack for compromising normal parasite neuromuscular activity.

## Supporting Information

S1 Fig The TRPV1 inhibitor SB 366791 does not significantly affect motility of *S*. *mansoni* adult worms. Shown are motility measurements for adult male (A) and female (B) worms exposed to 2 μM (black bars), 10 μM (cross-hatched bars), and 100 μM (white bars) SB 366791 vs. controls. Data are presented as means ± SEM. n = 7–8 for males, n = 4 for females.
(TIF)

S2 Fig RNAi suppresses expression of SmTRPA and SmTRPM7 RNAs. Shown are relative levels of SmTRPA or SmTRPM7 RNA determined by qRT-PCR, as described in Materials and Methods. **A.** SmTRPA knockdown. SmTRPA RNA levels are reduced in males by 90% and in females by 61% compared to control worms. **B.** SmTRPM7 knockdown. SmTRPM7 RNA levels are reduced in males by 42% and in females by 66% compared to control worms. **C.** SmTRPA/SmTRPM7 double knockdown. SmTRPM7 RNA levels are reduced in males by 71% and in females by 41%, and SmTRPA RNA levels are reduced in males by 88% and in females by 74%. n = 3 biological replicates for all data. Data are presented as means ± SEM.
(TIF)

S3 Fig Knockdown of SmTRPM7 alone does not alter adult worm responsiveness to capsaicin. Shown are motility responses to capsaicin in adult male (top) and female (bottom) *S*. *mansoni* electroporated with either luciferase (Luc) or SmTRPM7 (KD) siRNA. The only significant effect of SmTRPM7 knockdown appears to be in females exposed to 100 μM capsaicin, though even those worms still exhibit a ~20-fold increase in activity compared to no-drug controls. Concentrations of capsaicin are given below each condition. Data are presented as means ± SEM. *, P<0.05, unpaired t-test, n = 4–7.
(TIF)

S4 Fig Double knockdown of SmTPRA and SmTRPM7 reduces response to some concentrations of capsaicin compared to knockdown of SmTRPA alone. Comparison of motility responses of *S*. *mansoni* adults in which expression of SmTRPA or SmTRPA + SmTRPM7 (Double) has been suppressed by RNAi. Responses of male (left) and female (right) worms are shown. Data are presented as means ± SEM. *, ***, P<0.05, P<0.001, unpaired t-test, n = 4–7.
(TIF)

S5 Fig Knockdown of SmTRPM3a alters responsiveness of adult worms to both capsaicin and serotonin. Shown are motility responses of adult males (top) and females (bottom) to capsaicin (black bars) or 40 μM serotonin (5-HT; white bars). Worms were electroporated with either luciferase (Luc) or SmTRPM3a (KD) siRNA. Capsaicin concentrations are indicated. Note that knockdown of SmTRPM3a does appear to affect capsaicin sensitivity, but that it also results in significantly reduced responsiveness to serotonin, suggesting a general defect in the ability of worms to respond to agents that enhance worm motility. Data are presented as means ± SEM. n = 5–18 for males, 3–13 for females. *, **, ****, P<0.05, P<0.01, P<0.0001, unpaired t-test.
(TIF)

S1 Video Paired male and female adult schistosomes separate following exposure to capsaicin. A pair of adult male and female schistosomes was imaged at 2 frames per second over the course of 165 seconds following exposure to 100 μM capsaicin. Video plays at 30 frames per second, 15x faster than real time.
(MP4)

S2 Video Swimming behavior of *S*. *mansoni* cercariae before exposure to 60 μM capsaicin. Cercariae were imaged at 2 frames per second over the course of 150 seconds.
(MP4)

S3 Video Swimming behavior of *S*. *mansoni* cercariae in 60 μM capsaicin. The same worms as in S2 Video, beginning ~2.5 minutes following exposure to capsaicin. Note that though the cercariae still exhibit motility, they remain largely in a single location, without swimming about.
(MP4)
