# Supplementary figures and images for: Evidence for Novel Pharmacological Sensitivities of Transient Receptor Potential (TRP) Channels in Schistosoma mansoni
Source: PLoS Negl Trop Dis. 2015 Dec 11;9(12):e0004295. doi: 10.1371/journal.pntd.0004295 (PMC4676680; doi:10.1371/journal.pntd.0004295)

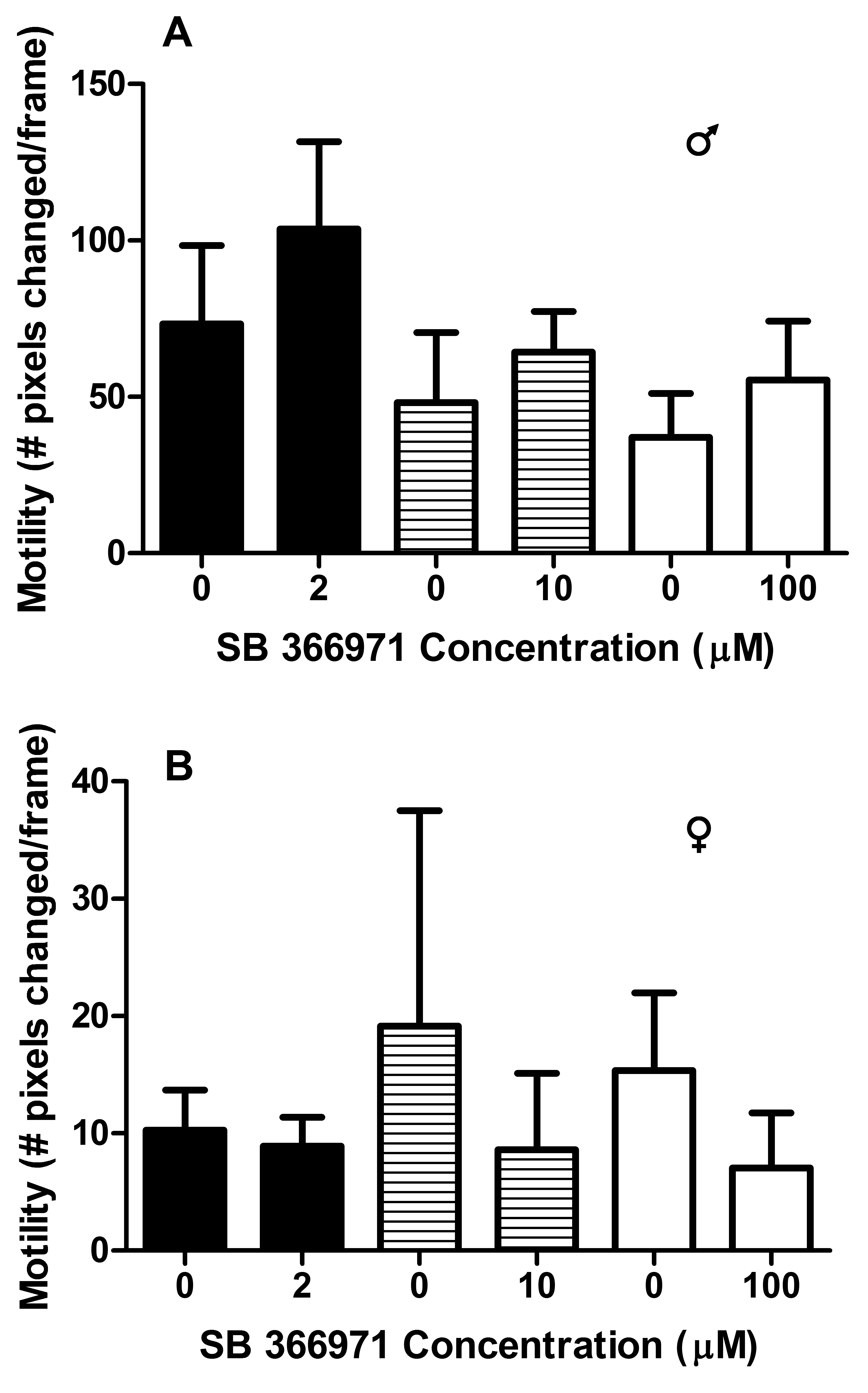

Supplement: S1 Fig — Shown are motility measurements for adult male (A) and female (B) worms exposed to 2 μM (black bars), 10 μM (cross-hatched bars), and 100 μM (white bars) SB 366791 vs. controls. Data are presented as means ± SEM. n = 7–8 for males, n = 4 for females. (TIF) [file pntd.0004295.s001.tif]

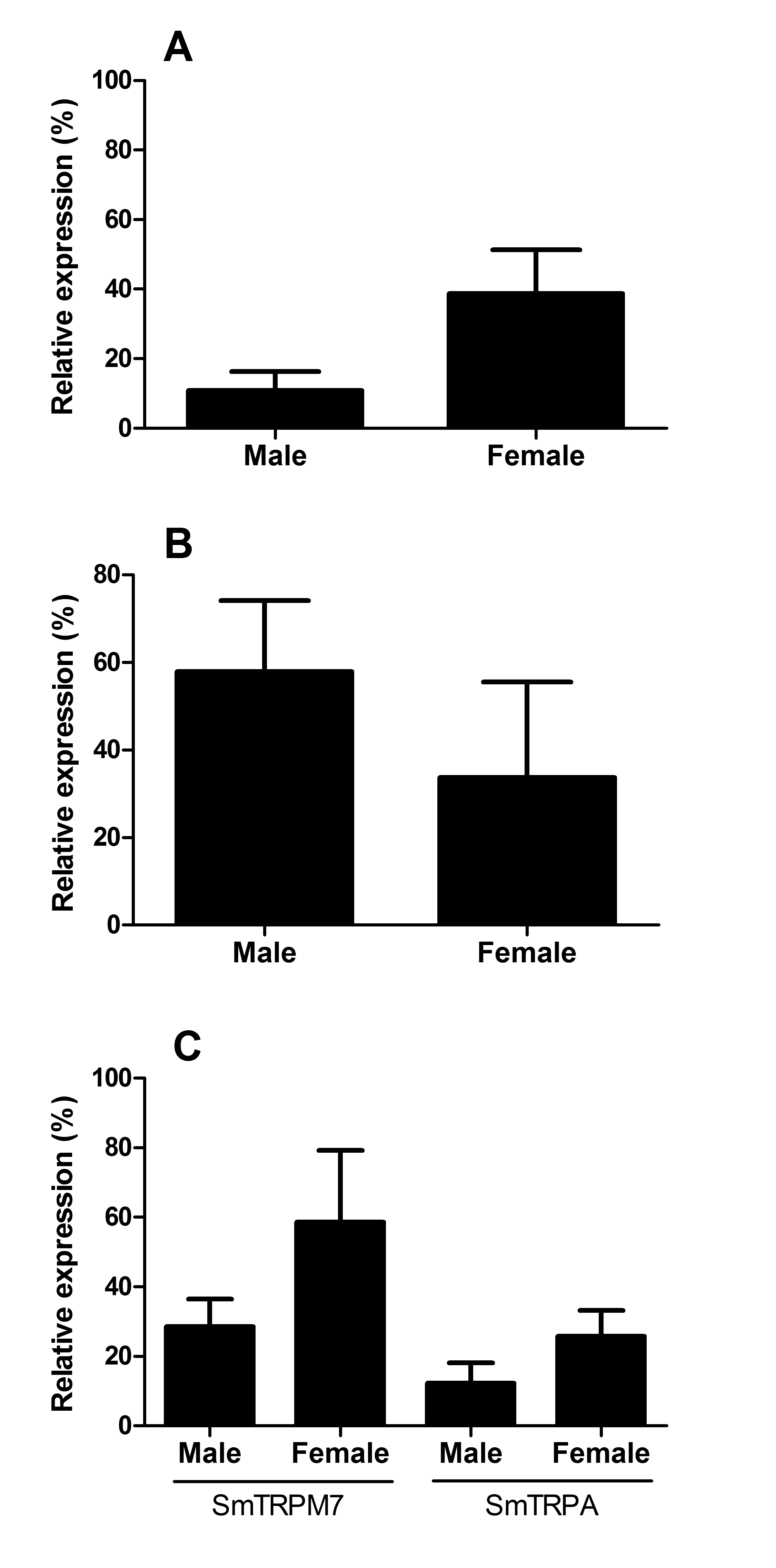

Supplement: S2 Fig — Shown are relative levels of SmTRPA or SmTRPM7 RNA determined by qRT-PCR, as described in Materials and Methods. A. SmTRPA knockdown. SmTRPA RNA levels are reduced in males by 90% and in females by 61% compared to control worms. B. SmTRPM7 knockdown. SmTRPM7 RNA levels are reduced in males by 42% and in females by 66% compared to control worms. C. SmTRPA/SmTRPM7 double knockdown. SmTRPM7 RNA levels are reduced in males by 71% and in females by 41%, and SmTRPA RNA levels are reduced in males by 88% and in females by 74%. n = 3 biological replicates for all data. Data are presented as means ± SEM. (TIF) [file pntd.0004295.s002.tif]

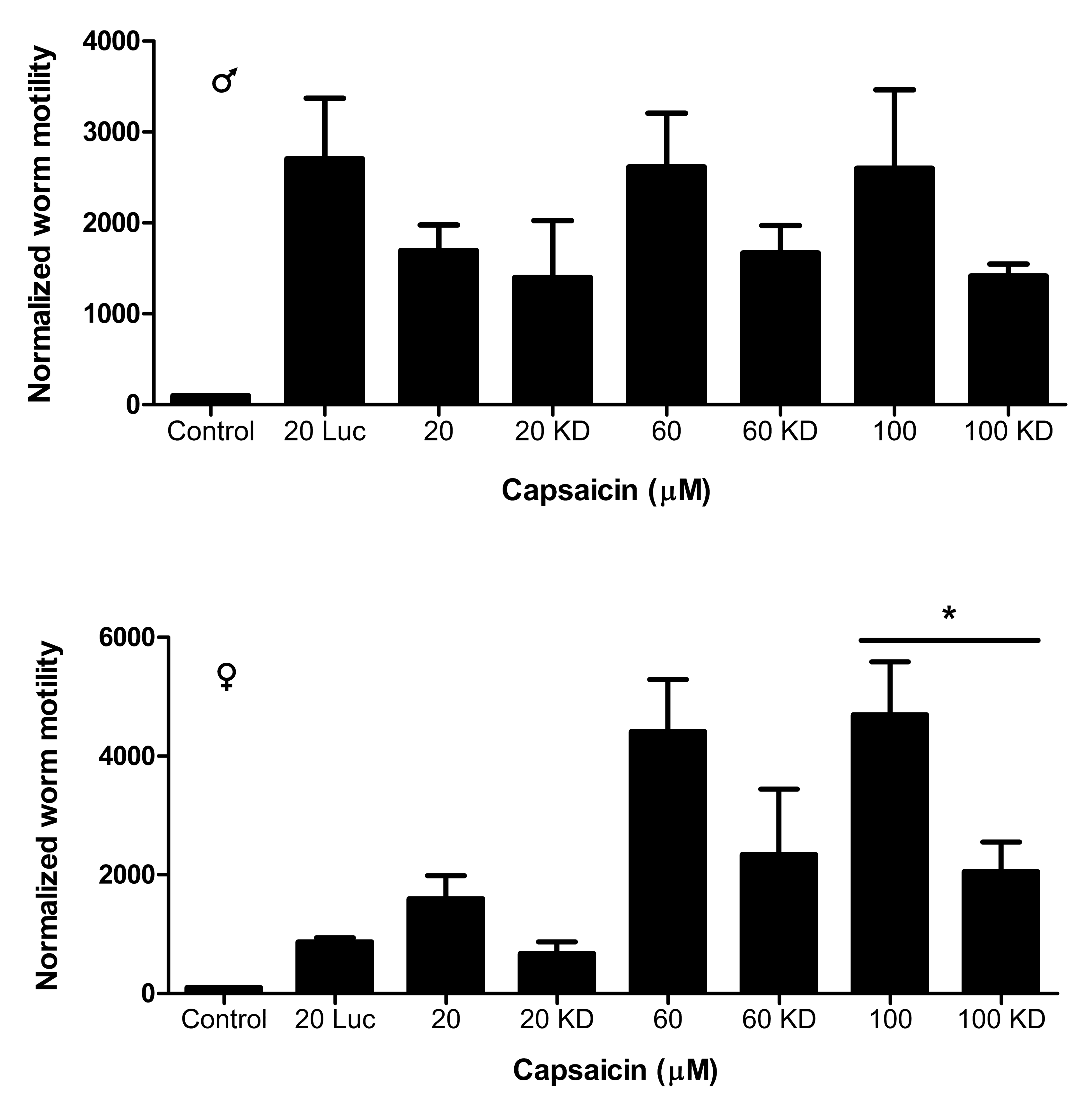

Supplement: S3 Fig — Shown are motility responses to capsaicin in adult male (top) and female (bottom) S. mansoni electroporated with either luciferase (Luc) or SmTRPM7 (KD) siRNA. The only significant effect of SmTRPM7 knockdown appears to be in females exposed to 100 μM capsaicin, though even those worms still exhibit a ~20-fold increase in activity compared to no-drug controls. Concentrations of capsaicin are given below each condition. Data are presented as means ± SEM. *, P<0.05, unpaired t-test, n = 4–7. (TIF) [file pntd.0004295.s003.tif]

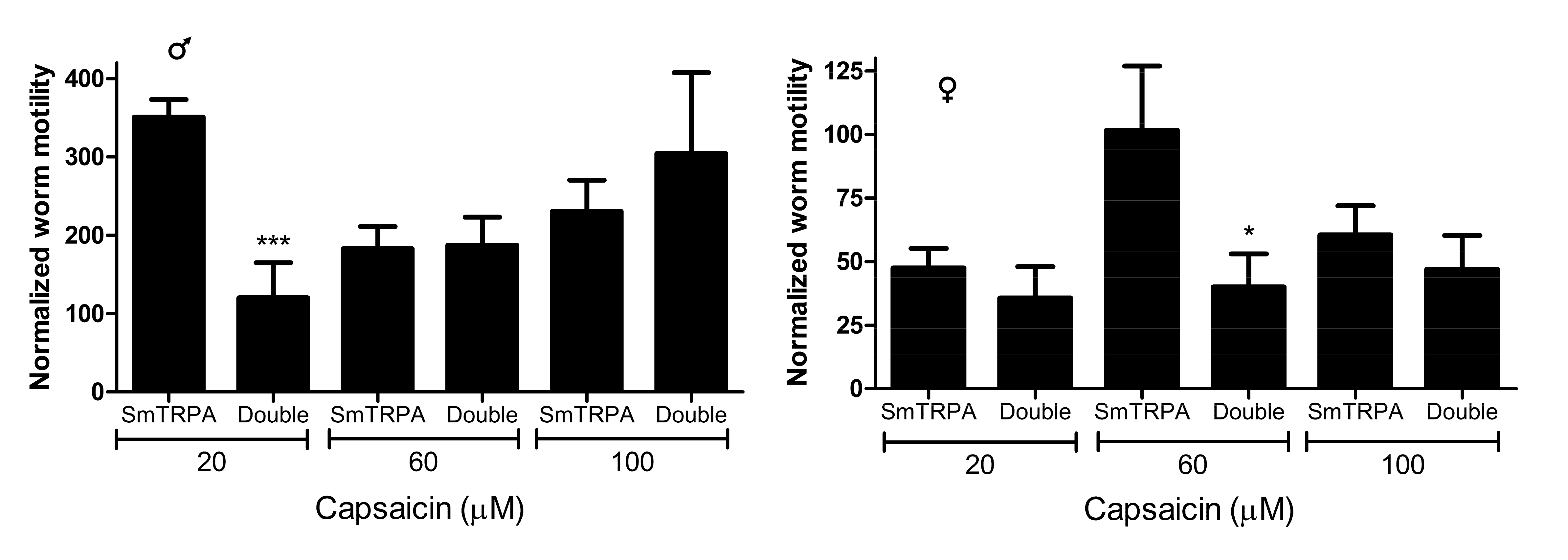

Supplement: S4 Fig — Comparison of motility responses of S. mansoni adults in which expression of SmTRPA or SmTRPA + SmTRPM7 (Double) has been suppressed by RNAi. Responses of male (left) and female (right) worms are shown. Data are presented as means ± SEM. *, ***, P<0.05, P<0.001, unpaired t-test, n = 4–7. (TIF) [file pntd.0004295.s004.tif]

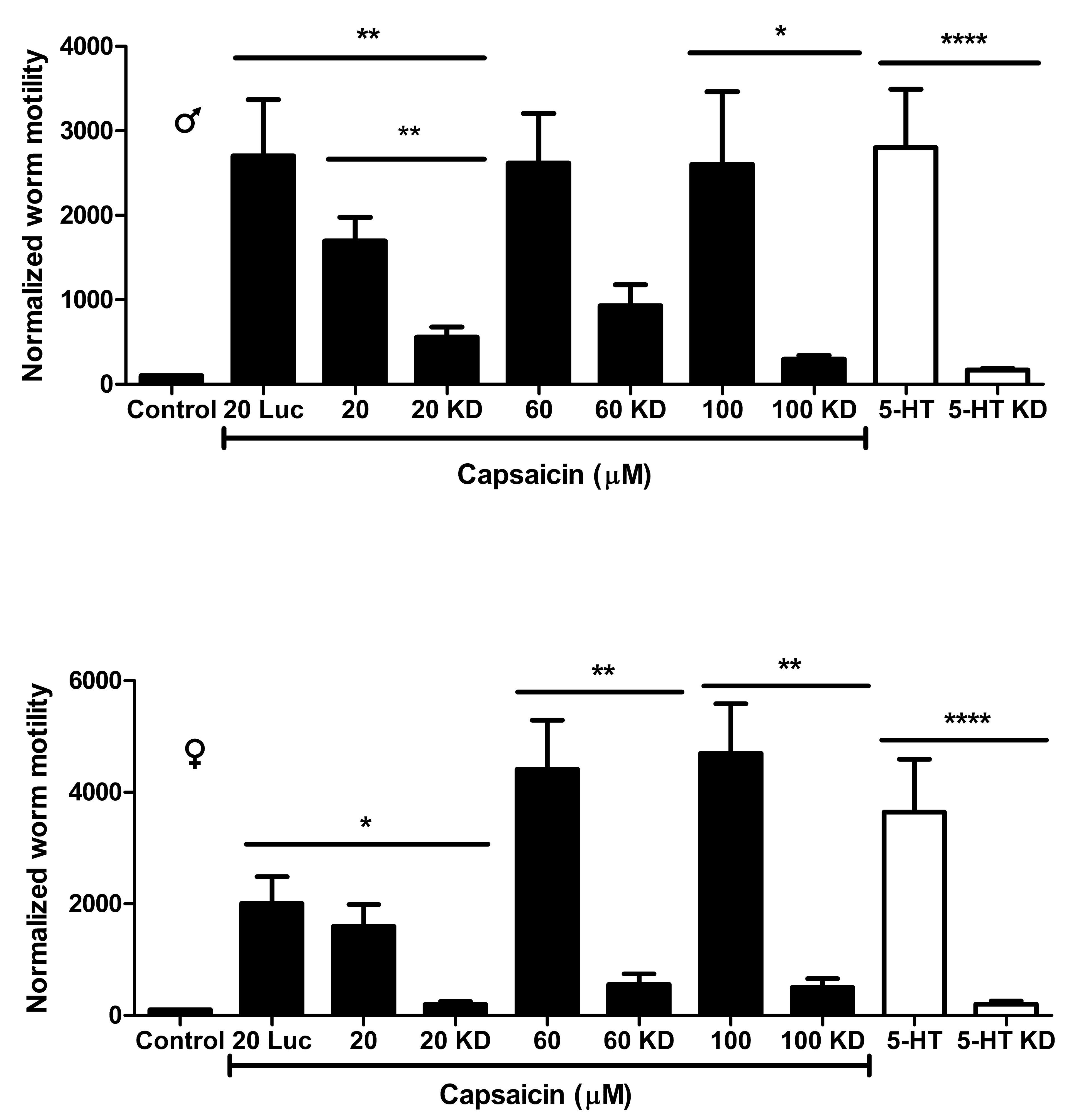

Supplement: S5 Fig — Shown are motility responses of adult males (top) and females (bottom) to capsaicin (black bars) or 40 μM serotonin (5-HT; white bars). Worms were electroporated with either luciferase (Luc) or SmTRPM3a (KD) siRNA. Capsaicin concentrations are indicated. Note that knockdown of SmTRPM3a does appear to affect capsaicin sensitivity, but that it also results in significantly reduced responsiveness to serotonin, suggesting a general defect in the ability of worms to respond to agents that enhance worm motility. Data are presented as means ± SEM. n = 5–18 for males, 3–13 for females. *, **, ****, P<0.05, P<0.01, P<0.0001, unpaired t-test. (TIF) [file pntd.0004295.s005.tif]
